# Correction: Offering Mental Health Services in a Conflict Affected Region of Pakistan: Who Comes, and Why?

**DOI:** 10.1371/journal.pone.0103700

**Published:** 2014-07-21

**Authors:** The PLOS ONE Staff

## Notice of Republication

This article was republished on July 3rd, 2014, due to Figure 1 and Figure 2 being ordered incorrectly. Please download the PDF again to view the corrected article. The originally published, uncorrected article and the republished, corrected article are provided here for reference.

## Supporting Information

File S1 Originally published, uncorrected article.
(PDF)

File S2 Republished, corrected article.
(PDF)
