# Supplementary material for: Correction: Offering Mental Health Services in a Conflict Affected Region of Pakistan: Who Comes, and Why?
Source: PLoS One. 2014 Jul 21;9(7):e103700. doi: 10.1371/journal.pone.0103700 (PMC4105562; doi:10.1371/journal.pone.0103700)
Supplement: File S1 — Originally published, uncorrected article. (PDF) [file pone.0103700.s001.pdf]

OPEN ACCESS Freely available online

PLOS | ONE

Offering Mental Health Services in a Conflict Affected Region of Pakistan: Who Comes, and Why?

Safieh Shah[1]*, Rafael Van den Bergh[2], Benedicte Van Bellinghen[3], Nathalie Severy[3], Sana Sadiq[4], Sher Ali Afridi[4], Asma Akhtar[4], Jacob Maïkére[3], Catherine Van Overloop[3], Saeed-ur-Rehman[5], Tahir Bashir-ud-Din Khilji[5], Saleem-ur-Rehman[5], Johan van Griensven[6], Serge Schneider[7], Philippe Bosman[3], Erwin Lloyd D. Guillergan[1], Francesca Dazzi[3], Rony Zachariah[2]

1 Medical Department, Médecins Sans Frontières – Operational Centre Brussels, Islamabad, Punjab, Pakistan, **2** Operational Research Unit (LuxOR), Médecins Sans Frontières – Operational Centre Brussels, Luxembourg, Luxembourg, **3** Medical Department, Médecins Sans Frontières – Operational Centre Brussels, Headquarters, Brussels, Belgium, **4** Mental Health Clinic, Médecins Sans Frontières– Operational Centre Brussels, Timurgara, Khyber Pakhtunkhwa, Pakistan, **5** Pakistan Ministry of Health (MoH), District Headquarter Hospital, Timurgara, Khyber Pakhtunkhwa, Pakistan, **6** Institute of Tropical Medicine, Antwerp, Belgium, **7** Médecins Sans Frontières – Operational Centre Luxembourg, Headquarters, Luxembourg, Luxembourg

Abstract

Background: North West Pakistan is an area ravaged by conflict and population displacement for over three decades. Recently, drone attacks and military operations have aggravated underlying mental disorders, while access to care is limited. Among patients attending a mental health clinic integrated in district hospital conducted by psychologists; we describe service utilization, patient characteristics, presenting complaints, morbidity patterns, and follow-up details.

Methodology/Principal Findings: A retrospective study using routinely collected programme data was conducted from February to December 2012. A total of 1545 consultations were conducted for 928 patients (86% females). There were 71(8%) children and adolescents. An increase was observed from February to July, followed by a decline. 163 new patients (18%) were on psychotropic medication at presentation. The most common morbidity in females (36%) were symptoms of adjustment disorders and acute reactions. Depression and anxiety were common in both genders while post traumatic disorder was frequent in males (21%). Out of the 928 new patients, 639(69%) had a follow up visit planned with their psychologist, but only 220(34%) new patients returned for a follow up visit.

Conclusion: In a district hospital, mental health services managed by psychologists were well attended. There is a need to consider widening the current package of care to cater to the diversity of mental health disorders, gender difference, children and adolescents. Standardized diagnostic and monitoring tools would also need to be adapted accordingly and to assess patient progress. Innovative approaches to tackle the problem of the low return rate are needed.

Citation: Shah S, Van den Bergh R, Van Bellinghen B, Severy N, Sadiq S, et al. (2014) Offering Mental Health Services in a Conflict Affected Region of Pakistan: Who Comes, and Why? PLOS ONE 9(6): e97939. doi:10.1371/journal.pone.0097939

Editor: Robert Stewart, Institute of Psychiatry, United Kingdom

Received January 28, 2014; **Accepted** April 26, 2014; **Published** June 25, 2014

Copyright: © 2014 Shah et al. This is an open-access article distributed under the terms of the Creative Commons Attribution License, which permits unrestricted use, distribution, and reproduction in any medium, provided the original author and source are credited.

Funding: This research was supported through an operational research course, which was jointly developed and run by the Operational Research Unit, Médecins Sans Frontières, Brussels Operational Centre, Luxembourg (LUXOR); the Centre for Operational Research, International Union Against Tuberculosis and Lung Disease (The Union), Paris, France and The Union South-East Asia Regional Office, Delhi, India. Additional support for running the course was provided by the Institute for Tropical Medicine, Antwerp, Belgium; the Center for International Health, University of Bergen; the University of Nairobi, Kenya and Partners In Health, Rwanda. This course is under the umbrella of the World Health Organization's special Programme for Research and Training in Tropical Diseases (WHO-TDR) SORT-IT program me (Structured Operational Research and Training Initiative) for capacity building in low- and middle-income countries. Funding for the course was provided by Médecins Sans Frontiéres Luxembourg, Brussels Operational Centre, Luxembourg, the Bloomberg Philanthropies and the Department for International Development (DFID), UK. There are no commercial funders and the funders had no role in study design, data collection and analysis, decision to publish, or preparation of the manuscript.

Competing Interests: The authors have declared that no competing interests exist.

* Email: msfocb-islamabad-med@brussels.msf.org

Introduction

A "mental disorder " or "illness" is defined as a psychological pattern, reflected in behavioural changes generally associated with distress or disability, and which is not considered part of normal development in a person's culture [1]. Mental disorders are a global problem as shown by surveys from 17 countries, which revealed a prevalence rate of 18–36% in the general population [2]. An estimated 13% of the global burden of disease is now attributed to mental disorders [3], with about 450 million people affected. The treatment gap for mental illness is about 90% in developing countries [4]. It is well-documented that conflict and displacement aggravate the pre-existing mental health burden of populations [5]. Mental health can be affected directly as well as indirectly by prolonged violence and displacement through, amongst others, direct experience of violence, disruption of societal structure, and reduced access to care [6]. The development of models for provision of mental health care in such settings is highly challenging, and few documented models of mental health care in developing countries [7] as well as conflict settings exist [8].

Although there is a dearth of research accurately measuring the burden of mental illness within Pakistan, according to one report approximately 10–16% of the population suffers from mental disorders [9]. The burden has been increasing over recent years and varies depending on the context – rural or urban [9,10,11]. In addition, there has been very limited investment in mental health care, further compounded by a dire shortage of skilled workers [12]. The interplay between conflict/displacement and mental health has been particularly striking in the North West province of Pakistan (Khyber Pakhtunkhwa – KPK) bordering Afghanistan, which for the past three decades has been beset by continuous violence [13]. Since 2001, the situation was aggravated by the invasion of Afghanistan by the United States of America (USA) followed by the USA's drone strikes. Military operations by the Pakistani army have also escalated in the same area. This has resulted in large movements of Internally Displaced Persons (IDPs), refugees and militants [14].

Provision of mental health care is challenging in Pakistan, and particularly in KPK care is usually provided as a centralised (health facility-based) service for security reasons, carrying certain access challenges. Anecdotal evidence suggests that acceptability is compromised by the perception that mental illness is not a medical problem, but one related to supernatural causes [15]. Overall, there is a lacuna in published literature on mental health needs from populations in northwest Pakistan[13,16,17]. Since 2012, the international non-governmental organisation Médecins Sans Frontières (MSF), in collaboration with the Pakistani Ministry of Health (MoH), provides mental health care as part of an integrated package of emergency medical assistance [8] in a district headquarter hospital, in the semi-urban setting of Timurgara in KPK. This model of integrated care relies solely on counselling by psychologists as first line mental health care providers. This is an attempt to use task-shifting to efficiently tackle the burden of cases for the inundated few, highly trained psychiatrists in Pakistan and in the future provide complementary psychological care (counselling) to patients also receiving psychotropic medication from general practitioners [18]. Information on utilization of mental health services, who actually accesses these services and for what condition, is useful to identify needs and orient programme policy and practice [19,20].

Among patients attending a mental health clinic in a conflict affected region in Pakistan, we thus describe a) the trend in service utilization, b) who is seeking care c) presenting complaint classified as reasons for seeking mental healthcare d) the morbidity and severity pattern and e) patients who returned for at least one follow-up visit.

Methods

Ethics Statement

This study has been approved by the Medical Research Council of Pakistan. The study has satisfied the Médecins Sans Frontières Ethics Review Board (Geneva, Switzerland) criteria for studies using routinely collected data. It has also been approved by the Ethics Advisory Group of the International Union Against Tuberculosis and Lung Disease, Paris, France. As the study used routine and aggregated data that was anonymized and de-identified prior to analysis, the issue of informed consent was not applicable.

Study Design

A descriptive, retrospective, cross-sectional study based on routinely collected patient data.

Study setting

The study was conducted in Timurgara district hospital, which lies in North West Pakistan, within Lower Dir, which is one of the 25 districts of the KPK province, bordering Afghanistan. KPK has about 20 million inhabitants, with a population density of 300/ km^2.

The health system suffers from a lack of drugs, equipment, and skilled workers. Health care in private clinics is on a payment basis and often unaffordable. Numerous checkpoints and military occupation of some public hospitals and clinics further compromises access to health care in the Lower Dir district [21].

In addition to the conflict-related background of KPK, there has also been a breakdown of infrastructures and considerable economic hardships [13]. This region lies in one of Pakistan's least developed areas, which has also seen the rise of militants and armed outfits.

Timurgura district hospital

MSF started an emergency medical assistance programme in Timurgara district hospital in 2010, which has approximately 350 beds. The mental health clinic was initiated in early 2012. The hospital is meant to cater to the inhabitants of Lower Dir (where Timurgara is located), but patients also come from all the neighbouring districts (Bajaur, Maidan in Lower Dir or Kohistan and Upper Dir).

The hospital offers a package of services including 1) general outpatient (MoH) and inpatient services (MSF with MoH) and emergency care (MSF with MoH), 2) specific care for mass casualties related to the ongoing violence (MSF), 3) comprehensive emergency obstetric and child care (MSF with MoH), 4) mental health care (MSF), and 5) health promotion (MSF). All services are provided free of charge.

The Mental Health Service package

The trigger factor for initiating mental health services in Timurgara hospital was the volume of patients seen in the emergency department (ED) diagnosed with serious mental disorders and for whom care was not available.

At the start of the programme and on a continuing basis, awareness raising campaigns on the existence of the mental health clinic were provided within the community [22] and the hospital to give people "psycho-education" sessions – identifying the importance of mental health. Patients in waiting areas of the hospital and the inpatient wards were also informed about such services through posters and leaflets for patients to get visuals in order to conceptualize psycho-education.

The guiding principles regarding mental health counselling within MSF include an integrated medical-psychological-social approach. Instead of focusing solely on a patient's symptoms, this counselling aims for the restoration of a patient as a functioning individual by identifying the patient's needs. Functionality is restored by utilizing coping mechanisms within a community-based culture (community support), available services (psychologists and/or psychiatrists), cultural and political-security as well as available human resources. This type of supportive counselling is provided within the frame of brief therapeutic approaches, and are expected to be culturally adaptable, based on the client's own objectives and resources and not on predetermined goals. However, techniques vary according to the academic background of the psychologists.

The psychologists are all Masters and Post-Masters graduates from Pakistan; holding national accreditation and experience in clinical psychology and therapeutic authorization as well. Further they are also repeatedly given training in accordance with MSF

standards and based on identified needs, and are regularly supervised by an expatriate clinical psychologist. All services are offered according to MSF standard guidelines.

Individuals who arrive at the mental care clinic are registered by said psychologists, and a patient file is opened. An assessment of their mental condition and its severity, including discussion of their main problems (presenting complaints), is then conducted by the psychologist. Consultations are conducted in two separate rooms by three national staff psychologists, which include two women and one man, since a considerable proportion of the caseload involves women. The patient is asked to identify the most important reason they sought care. This is termed the patient's "reason for coming" which is standardized (**Table 1**) according to MSF guidelines. Morbidity is graded on the basis of a "group of symptoms" which has case definitions (**Table 2**). This grading is based on the latest version of a set of standardized criteria utilized by Psychiatrists termed the Diagnostic and Statistical Manual of Mental Disorders (DSM-IV-TR) [23] for the classification of mental disorders. Although 205 different diagnoses are present in the DSM-IV-TR, the morbidity used by the psychologists in Timurgara are a condensed, simplified version, also used in other MSF mental health interventions in similar settings [24].

Patients requiring psychiatric care (including specific medications) were referred externally (to an MoH general practitioner in the OPD or an MoH hired psychiatrist), but these referral options only became available from December 2012 onwards.

Study population

The study included all individuals seeking mental health care from February 17th 2012 to December 31st 2012.

Data and analysis

Due to the retrospective nature of this study, informed consent was not taken, however patient records were anonymised and de-identified prior to analysis. A standardized mental health database was implemented in the programme, data from the patient files was single-entered into this database by the psychologists themselves, who were trained in data entry. The databases were regularly cross-checked on-site and also by the medical data team both in coordination office (Islamabad) and at headquarters (Brussels). Loss to follow up was defined as any patient who did not return for a planned first follow up visit anytime during a period of six months after the first consultation. Severity of the mental health condition was assessed using a Scale of Severity (1–10), which was introduced on the 2nd of May 2012 following the training of the counsellors. Patients were asked to look at a series of faces (**Figure 1**) and choose which one they identified with in terms of their perceived severity of the problem. It was used on a trial basis for the first consultation as well as for each subsequent follow up. There is however no scientific validation of this use of the tool.

Comparison between proportions was performed by Chi-square test. Differences in risk levels were assessed using relative risks (RR). The level of significance was set at <0.05. All analysis was done using EpiData Analysis (version 2.2.2.178, EpiData Association, Odense, Denmark).

Table 1. Patients reasons for seeking mental health care according to standardized guidelines[1] in Timurgara district hospital, Pakistan (2012).

1.	*Sadness/depressive mood:* when patient's reason for coming is about feeling sad, crying a lot, feeling depressed, having a low mood
2.	*Agitation/strong emotional reactions:* the patient is agitated, yelling, in a state of acute crisis or presenting an "hysterical" behaviour
3.	*Anxiety, Fear, Worry:* includes panic attacks, worrying a lot, feeling scared, heart beating fast, breathing difficulties or hyperventilation
4.	*Physical symptoms of stress/unexplained somatic complaints:* patient comes with somatic complaints: headache, palpitations, breathing difficulties, body pain
5.	*Traumatic experience:* when the patient seeks help or is referred following a traumatic experience related to a natural disaster, an accident or acts of violence
6.	*Sexual Violence:* the patient has been victim of or is suspected to have been victim of forced sexual acts carried out by civilians or armed actors
7.	*Severe Psychiatric:* when the client comes because of suffering from a severe psychiatric disorder such as schizophrenia, psychosis, manic depressive disorder or other where the patient is not in control or aware of own actions or suffers a disturbed sense of reality
8.	*Sleeping problem:* insomnia is the main problem the patient comes for
9.	*Substance abuse*: the client seeks help for using drugs or alcohol
10.	*Brain Dysfunction:* includes mental retardation, dementias, severe learning difficulties, epilepsy, personality/memory difficulties following stroke or blow to head and so on
11.	*Suicidal attempt:* the client has made a recent suicide attempt
12.	*Domestic/family Violence:* if the main problem is violence within the family, directed towards adults or children
13.	*Problem in Family/education of children:* if the main problem is related to marital problems/tensions, or problems with own children (education for example) or other family members, excluding the use of violence
14.	*Practical problem/socio economical:* includes economical, unemployment or other practical issues such as lack of shelter, food, means of transport, schooling
15.	*Displacement:* difficulties related to being displaced is the main reason for coming
16.	*Sexual/Reproductive health problem:* if main concerns are related to gender issues, sexuality, family planning, sexually transmitted infections, pregnancy, unwanted pregnancy, not having children and so on
17.	*Malnutrition:* Patient (or parents of patient) is referred or seeks help mainly related to a child malnutrition problem
18.	*Human immunodeficiency virus infection/acquired immunodeficiency syndrome*
19.	*Tuberculosis*
20.	*Other Medical:* the difficulties are related to a serious medical condition (chronic or acute) like long hospitalization, amputation, cancer and so on
21.	*Other:* other reason for patient seeking help or being referred not included above

[1]Medecins Sans Frontieres standardized mental health guidelines, Brussels Operational Centre, 94 Rue Dupre, Brussels, Belgium.
doi:10.1371/journal.pone.0097939.t001

Table 2. Grading[2] of mental health morbidity (case definitions) amongst individuals seeking care in Timurgara district hospital, Pakistan (2012).

1. Non-specific Symptoms	2. Symptoms of Anxiety disorders	3. Post Traumatic reactions	4. Symptoms of Depressive disorders	5. Symptoms of Psychotic disorders	6. Symptoms of Adjustment disorder/acute reactions	7. Behavioural Problems
Insomnia	Constant worry/anxiety	Intrusive thoughts of event	Sadness	Delusions	Recent stressful or traumatic event	Substance abuse
Loss of appetite	Restlessness	Flashbacks of event	Loss of interest	Hallucinations	Overwhelmed, feeling of being unable to cope	Aggressive behaviour
Low energy/weak	Fear	Nightmares of event	Hopelessness	Bizarre behaviour	Somatic symptoms	Delinquent behaviour/ acting out
Irritable/angry	Heart beating fast	Body reactions to reminders	No future plans	Disorganized thoughts	Anxiety/worry	Hyperactivity
Enuresis (children)	Pain in chest/heart	Avoiding reminders	Guilt	Disorganized speech	Low or sad mood	Withdrawal
Psychosomatic complaints	Breathing fast/difficulty	Overwhelming fear when reminded	Crying easily			Regression in development (children)
	Trembling	On guard	Suicidal thoughts			
	Always fearing the worst	Easily startled/jumpy	Absent feeling			
	Panic attacks	Repetitive games of event (children)	Feeling worthless			
	Phobia		Suicide attempt			

[2]Medecins Sans Frontieres standardized mental health guidelines, Brussels Operational Centre, 94 Rue Dupre, Brussels, Belgium.
doi:10.1371/journal.pone.0097939.t002

Results

Trend in service utilization

A total of 928 patients sought mental health care for the first time and underwent a total of 1545 mental health consultations during the study period. **Figure 2** shows the rising trend in service utilization soon after initiation of the mental health activities in the district hospital. A dip in the utilization trend was seen at the start of Ramzan (the month of fasting in the Islamic calendar, which lasted during July and August), which was followed by staffing problems in the programme.

Who is seeking mental health care?

Table 3 shows the patient socio-demographic characteristics, source of referral and history of psychotropic medication use. Patients were classified into age categories: patients of either gender most likely to attend mental health services fell between 19 to 45 years - 653 females (82%) and 96 males (76%). This was followed by patients between 13 to 18 years for males, although for females the second largest age group was of women above the age of 45 years.

There were a total of 71 (8%) individuals who were children and adolescents. 163 (18%) new patients were already on psychotropic medication at the time of first presentation to the clinic.

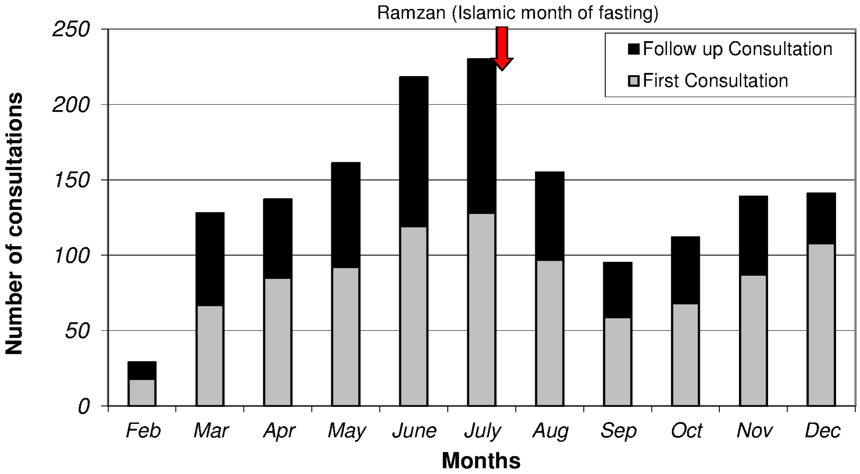

Figure 1. Scale of Severity. Separate pictorial scales, for both genders, used by the patient to score the magnitude of their mental health problem, assessed at their first mental health consultation, Timurgara district hospital, Pakistan (May - December 2012).
doi:10.1371/journal.pone.0097939.g001

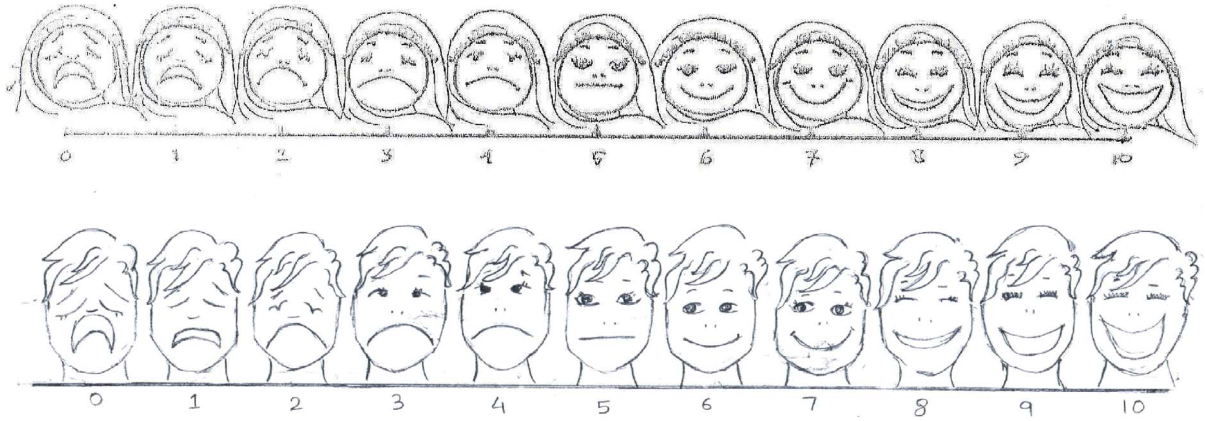

Figure 2. Trend in mental health consultations, Timurgara district hospital, Pakistan (February - December 2012).
doi:10.1371/journal.pone.0097939.g002

It is worth noting that although the number of mental health cases diagnosed in the ED ranged from 30 to 80 per month during the study period, only 35% of these went on to avail the mental health services.

Reasons for seeking mental health care and morbidity

Table 4 shows the reasons for seeking care (or presenting complaint) as expressed by the patient and **Table 5** the morbidity pattern as indicated by the psychologist. Considerable overlap

Table 3. Socio-demographic characteristics, source of referral and history of psychotropic medication use stratified by gender, Timurgara district hospital, Pakistan (2012).

	**Females N (%)**	**Males N (%)**
**Total**	**802 (100%)**	**126 (100%)**
**Age Group (years)**		
0–5	3 (0.4)	1 (0.8)
6–12	7 (0.9)	7 (5.6)
13–18	40 (5)	13 (10)
19–45	653 (81)	96 (76)
>45	99 (12)	9 (7.1)
**Referral Source**		
Inpatient Services	248 (31)	42 (33)
Outpatient Services	164 (20)	25 (20)
Community	156 (19)	52 (41)
**Health** promotion	142 (17)	6 (5)
Unknown	92 (12)	1 (1)
**Marital Status**		
Single	84 (10)	45 (36)
Spouse abroad	86 (11)	0
Married	596 (74)	68 (54)
Child	9 (1)	12 (10)
Widow/widower	22 (3)	1 (1)
Unknown	3 (0.4)	0
Separated/Divorced	2 (0.3)	0
**On Psychotropic Medication**		
Yes	116 (15)	47 (37)
No	660 (82)	71 (56)
Unknown	26 (3)	8 (6)

doi:10.1371/journal.pone.0097939.t003

Table 4. The main presenting complaints while seeking mental health care according to standardized guidelines[1] stratified by gender, Timurgara district hospital, Pakistan (2012).

Female (n = 802)		Male (n = 126)	
**Reason**	**N (%)**	**Reason**	**N (%)**
Anxiety, fear, worry	255 (32)	Anxiety, fear, worry	39 (31)
Sadness/depressive mood	215 (27)	Sadness/depressive mood	36 (29)
Sexual/reproductive health	158 (20)	Traumatic experience	24 (19)
Practical problems/socio-economic reasons	37 (5)	Sleeping problem	5 (4)
Agitation/strong emotional reaction	26 (3)	Substance abuse	3 (2)
Others*	111 (14)	Others*	18 (14)

[3]Medecins Sans Frontieres standardized mental health guidelines, Brussels Operational Centre, 94 Rue Dupre, Brussels, Belgium.
*This includes various other conditions listed in Box 1.
doi:10.1371/journal.pone.0097939.t004

exists between the presenting complaint and declared morbidity. The most common morbidities in females were adjustment disorders and acute reactions (36%). Depression and anxiety were common in both genders [25], while post traumatic disorder was frequent in men (21%) [26]. Findings showed that 15 out of 50 of the females who were children and adolescents exhibited adjustment disorders and acute reactions (30%), whereas for males within the same age group, (n = 21) it was post-traumatic reactions (n = 6, 29%).

For the assessment of severity, the Scale of Severity (1–10) was introduced on the 2nd of May, 2012. A total of 758 patients had their first consultation during this time, of which 87 were not scored, leaving a total of 671 patients of whom 88% were females and 12% were males. Self-reported severity was more diverse among males, and tended towards the more severe, self-reported, severity was more diverse among males, as well as more severe (Kruskal-Wallis $p<0.0001$) (**Figure 1 and 3**).

Patients who returned for follow-up visit(s)

Out of the 928 new patients, 639 (69%) had a follow up visit planned with their psychologist, of whom 220 (34%) actually returned to the clinic. Return rates for women were much lower than for men (31% versus 49% respectively, **Table 6**). Follow-up consultations were also conducted for 28 individuals who were not scheduled to return; a total of 617 follow up consultations were conducted for these 248 individuals (220+28).

Analysis of the interval between the initial consultation and the first follow up (**Table 6**) showed that while 85% of the female patients returned for their first follow up consultation within the first day of their initial consultation (as they were admitted at the hospital for other medical conditions), the figures drops steeply to only 12% returning from the second day onwards to the end of the first week. In contrast, males returning for their first follow up were more evenly distributed over time, with 29% coming back within the first day, 31% between the second day till the end of the first week, and finally, 27% coming back for their first follow up consultation within a month after their initial consultation.

When analysing the return rate (follow up consultation) in function of the service through which the patients were referred, the highest return rate was observed among patents referred from the inpatient department (52%), while it was worst for those referred by the mental health awareness raising team (5%) (**Table 7**).

Discussion

This study from a district hospital in a conflict affected area of Pakistan showed that mental health services were well used over their first year of operation. The age-groups were diverse, included children and adolescents and displayed distinct differences in male and female morbidity. Such diversity of patients, in terms of those seeking mental health, as well as their capacity to return for follow up, is challenging for any programme attempting to provide

Table 5. Mental health morbidity among new mental health consultations stratified by gender, Timurgara district hospital, Pakistan (2012).

Morbidity	Female N (%)	Male N (%)
**Total**	**802***	**126**
Non-specific Symptoms	37 (5)	11 (9)
Symptoms of Depressive Disorder	265 (33)	40 (32)
Post Traumatic Reactions	6 (1)	26 (21)
Symptoms of Anxiety Disorders	177 (22)	37 (33)
Symptoms of Psychotic Disorders	15 (2)	3 (2)
Symptoms of Adjustment Disorder/Acute Reactions	284 (36)	3 (2)
Behavioural problems	17 (2)	6 (5)

*The morbidity for 1 female patient was not recorded and therefore is missing data.
doi:10.1371/journal.pone.0097939.t005

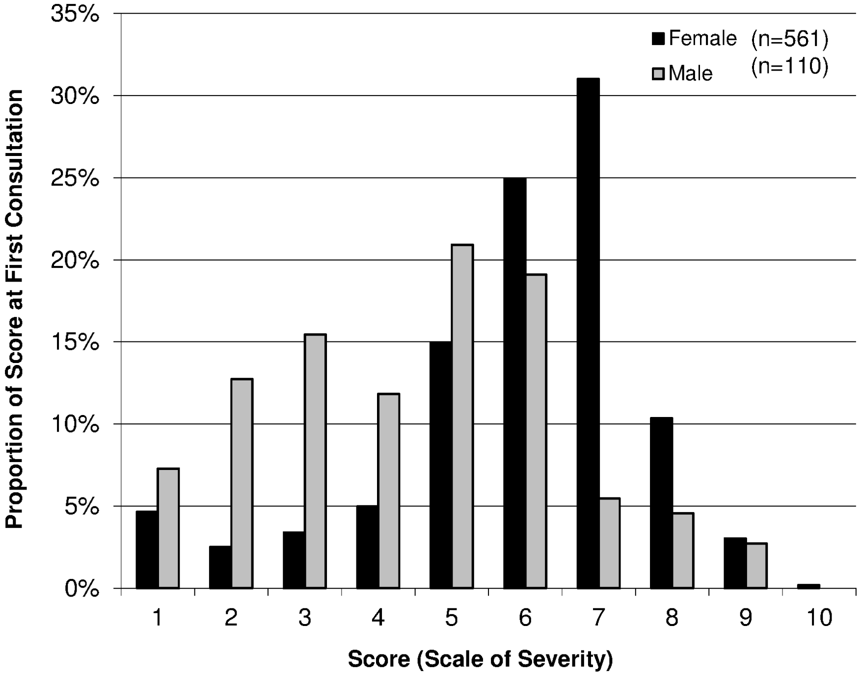

Figure 3. Score of patients. Patients (n = 671) whose Scale of Severity was assessed at their first mental health consultation, Timurgara district hospital, Pakistan (May - December 2012).
doi:10.1371/journal.pone.0097939.g003

standardised care using a generic approach [27]. The strengths of this study include the standardization of symptoms and morbidities, the use of clear case definitions, and the implementation of a specific mental health database to monitor the programme. There was also direct monitoring and evaluation of the data collected by the project (in Timurgara), and regular data entry training of psychologists, by the MSF coordination office in Islamabad. Although the severity scale was adapted to better suit the context of the patients; and their understanding of the scoring was also checked repeatedly, the reliability of this scale is yet to be ascertained. Psychologists in the programme are highly qualified, highly experienced and well trained as well as supervised. However given the diagnostic criteria, only one diagnosis termed as a group of symptoms was given per patient, representing a study limitation. Since the Severity Scale is not for the purposes of monitoring patient's progress (follow ups), further validation for this or other tools used within MSF will be required to measure the progress later on [8]. For a better understanding of the patient mental health severity pattern, tools to monitor therapeutic outcomes will be required, as currently the Severity Score does not seem to show any clear patterns of patient treatment evolution, either because the tool itself is not suitable for this purpose or because the effect of the intervention is difficult to quantify – the reason for this however, could not be identified at this stage. Finally, no assessment has been done to get an idea of what other health facilities or alternatives the patients utilize in the area, prior to, concurrently, or after they utilize the district hospital services; such information would help contextualize mental health care seeking habits [28].

A lesson learnt is that there are possible implications on the efficiency of the referral systems within the hospital services with regards to the low number of patients who actually show up to mental health services versus the number of those who are referred by these services within the hospital itself. Further, the majority of

Table 6. Follow up of mental health patients stratified by gender, Timurgara district hospital, Pakistan (2012).

	Female N (%)	Male N (%)
**Total (initial consultation)**	**802**	**126**
Follow up planned	526 (66)	113 (92)
Returning for first follow up (planned)	165 (31)	55 (49)
**Interval between initial consultation and first follow up**		
0–1 day	140 (85)	16 (29)
2–7 days	19 (12)	17 (31)
8–30 days	3 (2)	15 (27)
1–6 months	2 (1)	6 (11)
Unknown (incorrect/missing entry)	1 (0.6)	1 (2)

doi:10.1371/journal.pone.0097939.t006

Table 7. Associations between referral source and failure to return for a first follow up consultation among female patients attending mental health services, Timurgara district hospital, Pakistan (2012).

Referral source	First follow-up attended N (%)	No follow-up attended N (%)	Risk ratio (95% CI)	p-value
Total	220	419		
Inpatient service	121 (52)	108 (47)	1	-
Outpatient service	14 (15)	77 (85)	1.8 (1.5–2.1)	<0.0001
Community	22 (22)	76 (78)	1.6 (1.4–2.0)	<0.0001
Health promotion	5 (5)	88 (95)	2.0 (1.7–2.3)	<0.0001
Not recorded	3 (25)	9 (75)	NS	0.06

95% CI: 95% confidence interval; NS: non-significant.
doi:10.1371/journal.pone.0097939.t007

the patients who had a follow-up were inpatients and the psychologists visits the patient within the ward for follow up, rather than the patient coming to them to the mental health clinic.

During summer – May to August – there was an overall increase in patients attending the hospital services due to ease of access, versus during the winter (December to February) when the poorly constructed roads were blocked due to the snow. However due to Ramzan (Islamic month of fasting) which started at the end of July 2012, there was a drop of patients in August and September after which a gradual increase was again seen. The increasing utilisation of services at the district hospital points to the need to consider mental health services as a relevant minimum package of district hospital care in a conflict affected region [11]. Currently this is not considered part of the minimum WHO package of care for this health care facility level [29].

Of particular interest in this programme was that mental health clinics were managed by psychologists (and not psychiatrists), and this may represent a way forward towards task shifting and decentralisation of such services [30].

The caseload included children and adolescents. This group has age-specific counselling-related needs, which may require adaptation of the programme [24]. Furthermore, no specific training and skills were provided to the psychologists to cater to these groups and this will need to be addressed as well.

Further, three in ten patients who presented to psychologists were already on psychotropic medication without monitoring or evaluation by a psychiatrist or specialist or trained general practitioner. This points to poor drug regulation in developing countries like Pakistan, where medications are easily available without a prescription [31]. Given that no such drugs are part of the current package of care at the district hospital, there is an operational imperative to ensure referral of these patients on medication to specialized care.

In females, symptoms of adjustment disorders and acute reactions were the most common morbidity and could be related to marriage [32], in-laws [33], and the 'Dubai syndrome' [34] as well as other social pressures. Post traumatic reactions were common in males [26] and this may be a reflection of displacement and insecurity, which males are more exposed to within the area [35]. In terms of (self-assessed) severity of the condition, the majority of the females had a median score on the severity scale, while males showed more variability, with the scores more at the lower end of the scale. The score for females also ranged to include the high end, and could suggest that the services are frequented by less severe cases, or alternatively that the assessment tool is unable to capture the severity of the problem to a clear extent. Due to the high number of patients lost to follow up, the variability in time for follow-up, and the lack of validation of this Severity Scale as a follow-up tool, no analysis could be performed for the evolution of the Severity Scores over time. We thus call urgently for further research into establishing valid and relevant tools to evaluate the evolution of mental condition of the patient during the course of mental health care.

Return rates were very low, which was likely related to access constraints such as insecurity [13], centralisation of care, socio-cultural barriers [33], and the perception that mental illness is not a medical problem per se but one related to supernatural causes [36,37,38]. While the exact reasons for not returning for follow up could not be formally investigated in this study, we speculate here that the centralised nature of care in a district headquarter hospital may have played a role, as majority of the follow-ups were of patients admitted in the hospital, itself. Due to the status of the hospital as district referral hospital, patients typically come after initial screening at primary and secondary health services, and following extensive travel, pooling of resources from the family and community to organize transport, finding an attendant who is trustworthy and can translate as well as take care of the patient; and so on [39]. Such patients may have considerable difficulties in returning for follow up, in particular for conditions which are not perceived as being purely health problems [40,41].

This speculation is corroborated by the observation that patients (of both genders) who were admitted in the hospital for other medical illnesses were more likely to schedule follow ups with psychologists during the time they were inpatients. Such patients do not suffer from access barriers, as they are already on-site, or from social stigma concerning mental health, as they have been admitted for physiological or 'real' health problems. Additionally, the psychologists conducted the follow-up visits in the wards, actively seeking out their patients for the follow-up visit. This in contrast with patients who were given psycho-education sessions in the waiting areas by health promotion teams, who were unlikely to return for follow up.

This trend was particularly stark for female patients, who were most likely to fail to attend their first follow up session if the interval after the initial consultation went beyond one day. Amongst the small numbers whose psychologists did conduct follow ups with them, most were already admitted at the hospital. The majority of these cases were referred from the post-operative ward - the highest inpatient load in the hospital is of females who come for emergency obstetrical operative interventions or other Maternal and Child Health interventions.

Males, although more likely to return for their first follow up when admitted at the hospital, were also more likely to return after being discharged (as the hospital does not provide protracted or

long-term management). This could owe to their greater gender-based mobility in that cultural context – so they could travel alone and therefore avoid stigma associated with seeking mental health care. This is opposed to females who are not allowed to travel unaccompanied and without the permission of relatives.

Innovative ways for tackling these operational barriers are needed, which may include use of technology such as counselling via cellular phones or hotlines for follow up counselling [8]. Specific qualitative research into the determinants of losses to follow up could help guide such novel approaches [8,26]. Patients for whom follow up cannot in any way be ensured, the option for a Mental Health and Psychosocial support (MHPSS) single session exists especially within such a context [42]. However, this was not implemented in the programme as yet. The MHPSS expects that patients will be unable to return due to lack of access and security and the therapeutic strategy is different from the conventional approach [42].

In conclusion, mental health services being managed by psychologists in a district hospital in a conflict affected area in Pakistan are well attended. The findings of this feasibility study may provide a way forward as a model of care in such settings, but tailoring of the package of care will be needed and innovative approaches to ensure patient progress and monitor patient follow-up are urgently required.

Acknowledgments

This research was supported through an operational research course, which was jointly developed and run by the Operational Research Unit (LUXOR), Médecins Sans Frontières, Brussels Operational Centre, Luxembourg; the Centre for Operational Research, International Union Against Tuberculosis and Lung Disease (The Union), Paris, France and The Union South-East Asia Regional Office, Delhi, India. Additional support for running the course was provided by the Institute for Tropical Medicine, Antwerp, Belgium; the Center for International Health, University of Bergen; the University of Nairobi, Kenya and Partners In Health, Rwanda.

This course is under the umbrella of the World Health Organization (WHO-TDR) SORT-IT programme (structured operational research and training initiative) for capacity building in low- and middle-income countries. The input of the psychologists, including Qurat ul Ain Ismail and Mohammad Ibrahim are greatly appreciated. Further, the unwavering support of my peers, notably Syed Ali Ahmer Naqvi, as well as Ali Zubair, Maryiam Khawaja and the Addis and Kathmandu operational research cohorts was vital, without which this would never have come to fruition.

Author Contributions

Conceived and designed the experiments: S. Shah RVdB RZ JvG JM BVB S. Schneider JM PB CVO. Performed the experiments: NS BVB S. Sadiq SAA AA Saeed-ur-Rehman TBuDK Saleem-ur-Rehman. Analyzed the data: S. Shah RVdB RZ BVB NS FD. Contributed reagents/materials/ analysis tools: S. Shah RVdB NS BVB JM CVO Saeed-ur-Rehman TBuDK Saleem-ur-Rehman. Wrote the paper: S. Shah RVdB RZ JvG JM BVB S. Schneider PB CVO. Final approval of the version to be published: S. Shah RVdB RZ BVB S. Sadiq SAA AA NS ELDG JM FD Saeed-ur-Rehman TBuDK Saleem-ur-Rehman JvG S. Schneider PB CVO.

References

1. U.S. Department of Health and Human Services (1999) The fundamentals of mental health and mental illness. In: Department of Health and Human Services UPHS, editor. Mental health: a report of the Surgeon General. Rockville, USA: National Institute of Mental Health. pp. 32–116.
2. Kessler RC, Aguilar-Gaxiola S, Alonso J, Chatterji S, Lee S, et al. (2009) The global burden of mental disorders: an update from the WHO World Mental Health (WMH) surveys. Epidemiol Psichiatr Soc 18: 23–33.
3. WHO (2012) Global burden of mental health disorders and the need for a comprehensive, coordinated response from health and social sectors at the country level - 65th World Health Assembly resolution. Geneva, Switzerland: WHO.
4. WHO (2003) Investing in mental health. Geneva, Switzerland: WHO.
5. Baingana F, Bannon I, Thomas R (2005) Mental health and conflicts: conceptual framework and approaches. Washington, USA: World Bank Human Development Network.
6. Knapp M, Funk M, Curran C, Prince M, Grigg M, et al. (2006) Economic barriers to better mental health practice and policy. Health Policy Plan 21: 157–170.
7. Saraceno B, van Ommeren M, Batniji R, Cohen A, Gureje O, et al. (2007) Barriers to improvement of mental health services in low-income and middle-income countries. Lancet 370: 1164–1174.
8. Shanks L, Ariti C, Siddiqui MR, Pintaldi G, Venis S, et al. (2013) Counselling in humanitarian settings: a retrospective analysis of 18 individual-focused non-specialised counselling programmes. Confl Health 7: 19.
9. WHO (2011) Mental health atlas 2011. Geneva, Switzerland: WHO.
10. World Bank (2011) NCDs policy brief - Pakistan Washington, USA: World Bank Human Development Network.
11. Gadit AA (2007) Psychiatry in Pakistan: 1947–2006: a new balance sheet. J Pak Med Assoc 57: 453–463.
12. Siddiqi K, Siddiqi N (2007) Treatment of common mental disorders in primary care in low- and middle-income countries. Trans R Soc Trop Med Hyg 101: 957–958.
13. Khalily MT (2011) Mental health problems in Pakistani society as a consequence of violence and trauma: a case for better integration of care. Int J Integr Care 11: e128.
14. Husain N, Chaudhry IB, Afridi MA, Tomenson B, Creed F (2007) Life stress and depression in a tribal area of Pakistan. Br J Psychiatry 190: 36–41.
15. Gadit A, Reed V (2004) Culture and mental health: Pakistan's perspective. Karachi, Pakistan: Hamdard Foundation.
16. WHO, Ministry of Health Pakistan (2009) WHO-AIMS report on mental health system in Pakistan. Pakistan: WHO.
17. Irfan M, Anwar S, Raza UA, Qayum M (2011) Assessing the psychosocial support services for displaced persons in Pakistan based on Sphere standards and indicators Journal of Postgraduate Medical Institute 25: 118–126.
18. Kakuma R, Minas H, van Ginneken N, Dal Poz MR, Desiraju K, et al. (2011) Human resources for mental health care: current situation and strategies for action. Lancet 378: 1654–1663.
19. Demyttenaere K, Bruffaerts R, Posada-Villa J, Gasquet I, Kovess V, et al. (2004) Prevalence, severity, and unmet need for treatment of mental disorders in the World Health Organization World Mental Health Surveys. JAMA 291: 2581–2590.
20. Chisholm D, Sekar K, Kumar KK, Saeed K, James S, et al. (2000) Integration of mental health care into primary care. Demonstration cost-outcome study in India and Pakistan. Br J Psychiatry 176: 581–588.
21. Khalily MT, Fooley S, Hussain I, Bano M (2011) Violence, psychological trauma and possible acute post-traumatic interventions in Pakistani society. Australasian Journal of Disaster and Trauma Studies 1: 1–9.
22. Afridi MA (2008) Mental health: priorities in Pakistan. J Pak Med Assoc 58: 225–226.
23. American Psychiatric Association (2000) Diagnostic and statistical manual of mental disorders, fourth edition, text revision (DSM-IV-TR).
24. Llosa AE, Casas G, Thomas H, Mairal A, Grais RF, et al. (2012) Short and longer-term psychological consequences of Operation Cast Lead: documentation from a mental health program in the Gaza Strip. Confl Health 6: 8.
25. Mumford DB, Nazir M, Jilani FU, Baig IY (1996) Stress and psychiatric disorder in the Hindu Kush: a community survey of mountain villages in Chitral, Pakistan. Br J Psychiatry 168: 299–307.
26. Steel Z, Chey T, Silove D, Marnane C, Bryant RA, et al. (2009) Association of torture and other potentially traumatic events with mental health outcomes among populations exposed to mass conflict and displacement: a systematic review and meta-analysis. JAMA 302: 537–549.
27. Prince M, Patel V, Saxena S, Maj M, Maselko J, et al. (2007) No health without mental health. Lancet 370: 859–877.
28. WHO (2013) Mental health action plan 2013 - 2020. Geneva, Switzerland: WHO.
29. James S, Chisholm D, Murthy RS, Kumar KK, Sekar K, et al. (2002) Demand for, access to and use of community mental health care: lessons from a demonstration project in India and Pakistan. Int J Soc Psychiatry 48: 163–176.
30. Bolton P (2013) Mental health in Iraq: issues and challenges. Lancet 381: 879–881.
31. Khan MM (2006) Murky waters: the pharmaceutical industry and psychiatrists in developing countries. The Psychiatrist 30: 85–88.
32. Mirza I, Jenkins R (2004) Risk factors, prevalence, and treatment of anxiety and depressive disorders in Pakistan: systematic review. BMJ 328: 794.

33. Fisher J, Cabral de Mello M, Patel V, Rahman A, Tran T, et al. (2012) Prevalence and determinants of common perinatal mental disorders in women in low- and lower-middle-income countries: a systematic review. Bull World Health Organ 90: 139G-149G.
34. Ahmed SH, Zainulabdin F (1991) Dubai syndrome in Karachi. J Pak Med Assoc 41: 10–12.
35. Coldiron ME, Llosa AE, Roederer T, Casas G, Moro MR (2013) Brief mental health interventions in conflict and emergency settings: an overview of four Medecins Sans Frontieres — France programs. Confl Health 7: 23.
36. Gadit AA, Callanan TS (2006) Jinni possession: A clinical enigma in mental health. J Pak Med Assoc 56: 476–478.
37. Gadit AAM, Vahidy A (1999) Mental health morbidity pattern in Pakistan. Journal of College of Physicians and Surgeons Pakistan 9: 362–365.
38. Mubbashar MH (2003) Development of mental health services in Pakistan. International Psychiatry 1: 11–14.
39. Ali FA, Israr SM, Ali BS, Janjua NZ (2009) Association of various reproductive rights, domestic violence and marital rape with depression among Pakistani women. BMC Psychiatry 9: 77.
40. Gadit AAM (2007) Psychiatry in Pakistan: 1947–2006: A New Balance Sheet. Journal of Medical and Biological Science 1: 1–21.
41. Ahmed SH (2007) Mental Health: the impact of culture and diversity. Pakistan Association for Mental Health: 1–4.
42. Paul KE, Van Ommeren M (2013) A primer on single session therapy and its potential application in humanitarian situations. Interv Int J Mental Health, Psychos Work Couns Areas Armed Conflict 11: 8–23.
